# Electrical Tweezer for Droplet Transportation, Extraction, Merging and DNA Analysis

**DOI:** 10.3390/mi8120353

**Published:** 2017-11-30

**Authors:** Ali Shahid, Sylvia Chong, James Mahony, M. Jamal Deen, P. Ravi Selvaganapathy

**Affiliations:** 1 Department of Mechanical Engineering, McMaster University, 1280 Main St. W., Hamilton, ON L8S 4L7, Canada; saim_ali71@hotmail.com; 2 Regional Virology Laboratory, St. Joseph’s Healthcare Hamilton, Hamilton, ON L8N 4A6, Canada; sylviachong3@gmail.com (S.C.); mahonyj@mcmaster.ca (J.M.); 3 Department of Pathology and Molecular Medicine, McMaster University, Hamilton, ON L8S 4L8, Canada; 4 Department of Electrical and Computer Engineering, McMaster University, Hamilton, ON L8S 4L7, Canada; jamal@mcmaster.ca

**Keywords:** droplets, electrical tweezer, microfluidics, amplification, nanoliter, droplet extraction, droplet merging, droplet transportation

## Abstract

Droplets of aqueous solutions distributed in an immiscible oil phase are increasingly used and investigated as a means to handle and assay small volumes of samples. The primary attraction of this method is that surface interactions are kept to a minimum, and changes in sample concentration, especially due to adsorption to the walls, are avoided. Microfluidic methods to generate, transport, merge, split and perform reactions in droplets were developed recently. These methods depend on the continuous flow of the two phases involved inside closed microfluidic channels. Alternatively, an electrowetting phenomenon was also exploited to control the movement of droplets between two solid substrates. However, there are some situations where small volume sample transport and assaying are required in open systems. Here, we demonstrate a simple electromechanical probe (tweezers) that is capable of manipulating a small aqueous droplet in a bi-layer oil phase. The tweezer consists of two needles positioned close to each other and uses polarization of the aqueous droplet in an applied electrical field to confine the droplet between the needles with minimal solid contact. Mechanical motion of the tweezer can be used to transport the droplet to various positions. Operations such as aliquoting, merging and transport are demonstrated. Finally, this method was used to perform a DNA amplification assay where droplets of the sample and the amplification mixture are aliquoted separately, mixed and amplified using an in-situ heater. This electromechanical tweezer is of interest in low-throughput, small-volume biological and chemical assays where the investigator requires direct and open access to the samples.

## 1. Introduction

Droplet-based microfluidics has been increasingly investigated over the last decade, especially for performing small-volume assays in biology [1,2,3,4,5,6] and chemistry [7,8,9,10,11]. Some of the advantages of droplet-based methods over the conventional channel-based microfluidic systems are low surface adsorption of sample contents due to minimal surface contact, discretization of sample that enables multiple parallel assays and rapid processing time for bioassays due to small volumes. For high-throughput droplet assays, continuous-flow-based methods were developed to perform sample aliquoting, mixing, droplet merging, splitting and reaction using a continuous flow of an aqueous and an oil phase inside a closed microfluidic channel [12,13,14,15,16]. For low-throughput assays, methods that control or use the surface tension of individual droplets such as electro-wetting [17,18] and wire-guided systems [19] or those that use electric fields to transport charged droplets were developed [7].

One of the more established technology for droplet manipulation is digital microfluidics (DMF) [20]. In this method, discrete droplets between two insulating and hydrophobic substrates can be manipulated by spatial control of the contact angle of the substrate through electrical means. It uses the principle of electro-wetting on dielectric (EWOD) where the application of a potential on an electrode underneath a dielectric material changes the local contact angle of an aqueous droplet on it [17,21]. Thus, computer controlled manipulation of the electrode potentials in an array of electrodes placed adjacent to each other can be used to transport, mix, merge or break up droplets [22,23]. Although it is a versatile method, the need for fabrication of the electrode array and computer control of the potentials on many electrodes make this technique quite elaborate to implement in cases where single or a few droplets need to be manipulated and controlled in a simple fashion. In addition, due to the presence of substrates on the top and bottom, direct access to the manipulated droplet in order to either withdraw or infuse additional reagents is not straightforward [18].

Recently, a new digital microfluidic system has been introduced based on the principle of electrophoresis of a charged droplet (ECD) [24]. In this method, a discrete droplet suspended in an oil medium can be manipulated over the array of bare electrodes by electrical means. It uses the principle of droplet charging by the direct contact with a charged electrode to then electrophoretically move it along the electric field lines. By suitably manipulating the electrodes where the potential is applied, the electric field in the oil medium can be changed and the droplet transported [7]. Even though this is a low-cost fabrication technology and has demonstrated the ability to perform basic microfluidic operations such as mixing and coalescence [8,25], it requires a sophisticated computer control system to continuously switch the polarities between the electrodes. Furthermore, some unit operations such as aliquoting of precise volumes of fluid into droplets from a reservoir may be difficult using this technology.

An alternate method of using a wire-guided system to manipulate and transport individual droplets was recently developed [19]. In this arrangement, an aqueous droplet is placed on the chemically treated super-hydrophobic substrate or submerged in hydrophobic oil. A metal wire or a needle is used to attach and transport the droplet to various locations. The method was used to perform various microfluidic operations such as mixing and merging of droplets. It uses the principle that droplets have strong adhesive forces with the wire, which is relatively hydrophilic, as compared to the substrate/oil medium. Therefore, the droplet energetically prefers to remain attached to the wire and follow it as the wire is moved around. Even though this is a low-cost method, precise control of the wire is required to initially bring it in contact with the droplet and this system is difficult to implement where rapid transportation of water droplet is required as droplet may lose contact with needle [26,27,28]. Finally, it is difficult to detach the droplet from the wire for subsequent operations.

In many cases, a simple tweezer capable of handling and manipulating small droplets mechanically would be preferable. It should be an intuitive and easy method to visualize the droplet and direct its movement manually without the use of extensive software control. The tweezers themselves should be reliable in their ability to hold and release the droplets on-demand. In this paper, we introduce such a tweezer that uses the electric field to trap droplets between its fingers. This tweezer can be manually manipulated and provides an easy visual feedback of the motion that is intuitive to understand. We show that this electrical tweezer is capable of holding, releasing and transporting droplets, merging and mixing two droplets, as well as extracting droplets of precise volume from a reservoir. Finally, we demonstrate the use of this tweezer in a sample assay involving by loop-mediated isothermal amplification of DNA.

## 2. Design of the Electrical Tweezer

**Figure 1:**
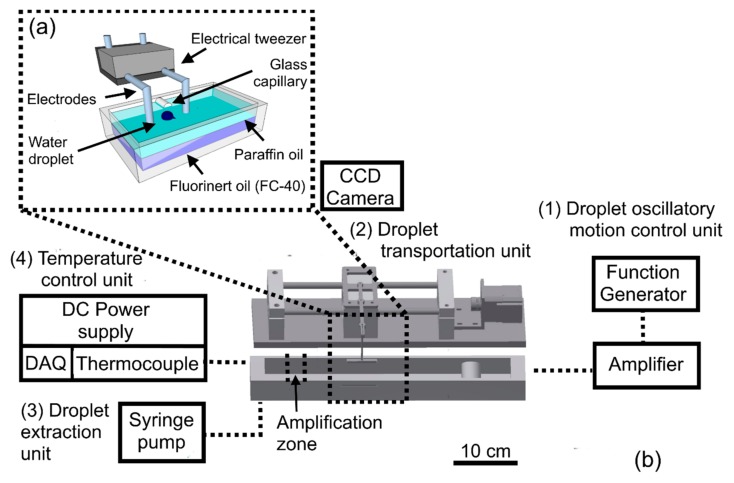
(**a**) Electrical tweezer system; (**b**) experimental setup to study the electrically influenced droplet motion, transportation, extraction, merging and DNA amplification through electrical tweezers.

The electrical tweezer was designed to mechanically handle the discrete water droplets between the dielectric bi-layer medium. It is composed of two main components: electrodes (fingers of the tweezer) and a holder (Figure 1a). The electrodes were made by mechanical bending of stainless steel tubes (diameter 0.01″, 304 Stainless Steel tubing, McMaster-Carr, Elmhurst, IL, USA), while the holder was fabricated using a 3D printer. These electrodes were immobilized in a holder to keep them at the fixed distance. The shape and distance between the electrodes are two important factors when building the electrical tweezer. Rod- or sheet-shaped electrodes could be used. While the sheet shaped electrodes provide a more uniform electric field distribution between them, they obstruct the view of the droplet and are also difficult to manipulate. Therefore, rod-shaped needle electrodes were chosen. The distance between the electrodes should be optimized such that it is large enough to accommodate and allow droplet motion while being small enough that the potential applied to generate the required electric field is small.

A bi-layer of dielectric liquid consisting of paraffin (Caledon Laboratories Ltd, Georgetown, ON, Canada) and Fluorinert oil (FC-40, 3M, St. Paul, MN, USA) was used to position the droplets in a single horizontal plane. The Fluorinert oil settled at the bottom due to its high density (1855 kg/m^3^), while paraffin oil (800 kg/m^3^) floated on top. A droplet of water dispensed via a pipette into the bilayer of oil naturally settled at the interface between the oils. The droplet was positioned between the electrodes before the start of the experiments. The fixed distance between the electrodes ranges from ~4 to 10 mm based on the droplet size that is to be handled.

## 3. Experimental Setup

The experimental setup (Figure 1b) mainly consists of four parts: (1) Droplet oscillatory motion control unit; (2) Droplet transportation unit; (3) Droplet extraction unit; and (4) Temperature control unit. The droplet oscillatory motion was visualized and recorded by a charge-coupled device (CCD) camera (Coolpix P6000, Nikon, Melville, NY, USA) mounted on a microscope. The electric potential was applied by a function generator (AFG 3022B, Tektronix, Inc., Beaverton, OR, USA) and amplified with a high-voltage amplifier (Trek 10/10B-HS, Trek, Inc., Lockport, NY, USA). One electrode of the tweezer was the source electrode (high-voltage electrode) while, the second was grounded. To characterize the droplet transportation, the electrical tweezer was mounted on the motorized linear stage (fabricated in-house) and was moved ~20 cm. The linear motorized stage was powered by direct current (DC) power supply (E3617A, Agilent Technologies, Inc., Santa Clara, CA, USA). Additionally, a syringe pump (KD Scientific, Inc., Holliston, MA, USA) was used only to inject water samples through the glass capillary (Microhematocrit Capillary Tubes, Fisher Scientific, Pittsburgh, PA, USA) at the interface of the two-oil medium to study the droplet extraction while, droplets were dispensed by the pipette in the oil medium for all other characterization experiments. Finally, a heating element, fabricated in-house using a resistive wire (NI80, Omega Engineering, Inc., Norwalk CT, USA), powered by the DC power supply (GPS-1850D, Good Will Instrument Co., Ltd., Taiwan) was used to establish the elevated temperature zone (~65 °C) in the oil chamber. The temperature was controlled by LabVIEW (V 14.0.0, National Instruments, Austin, TX, USA) with a PID module that was connected to the heat generation and data acquisition units through the DAQ (NI-6008, OMEGA Engineering, Inc., Houston, TX, USA). Thermocouple (TMQSS-020U-12, OMEGA Engineering, Inc., Houston, TX, USA), A signal conditioner (Omega-CCT) provided feedback to control the temperature.

### 3.1. Image Analysis

The oscillatory motion of droplet under a high electric field was studied by analyzing the recorded images. The sequential images (every 0.07 s) were recorded by the CCD camera analyzed by the ImageJ 1.51K (http://rsbweb.nih.gov/ij/). These images were used to measure the droplet position in each frame under a high electric field. To measure this distance, an image of a length scale (resolution 10 µm) was taken with the same imaging setup and was analyzed with ImageJ. The number of pixels corresponding to the known distance of the length scale was measured and marked as a reference distance. This reference distance was used to measure the oscillatory distances. The droplet position in each frame was determined by measuring the linear distance between the edge of the droplet and the high-voltage electrode. The maximum oscillatory distance of the droplet was the average of maximum distance of the droplet for 10 complete oscillatory cycles, and the standard deviation was plotted as error bars.

### 3.2. Loop-Mediated Isothermal Amplification (LAMP) Assays

Loop-mediated isothermal amplification (LAMP) assays used to detect the Shiga toxin gene *eae* virulence factor gene of *E. coli* (*Escherichia coli*) 0157 consisted of sets 5 or 6 of primers for the specified gene. Research use only (RUO) reagents required for LAMP were purchased from Canadian Molecular Developments (Division of Pro-Lab Diagnostics, Richmond Hill, ON, Canada). The LAMP reaction mixture (25 µL) consisted of three components, Optigene master mix (15 µL), primer mix for a specified gene (5 µL), and a DNA template (5 µL). Optigene master mix (ISO-0001 Isothermal Master Mix, Optigene, Horsham, UK) consisted of MgSO_4_, dNTPs, and ds-DNA binding dye. The primer mix for the targeted gene consisted of primer mix: forward outer (F3) and backward outer (B3) primers at 0.2 µM, forward inner (FIP) and backward inner (BIP) primers 0.8 µM, and forward loop (LF) and backward loop (LB) primers at 0.4 µM. For positive control, a DNA template (5 µL) was added to make a final concentration 10^8^ DNA/reaction. For negative reaction, the DNA template was replaced by ddH_2_O. The fluorescence reader (ESE-Quant TS95, Qiagen, Hilden, Germany) was used for both real-time nucleic acid amplification and end-point measurement of fluorescence intensity of samples treated by electrical tweezer.

## 4. Results and Discussions

The electrical tweezer was used to manipulate water droplets in a dielectric oil medium under various conditions. First, the droplet motion with the high voltage electrode in the dielectric medium was characterized under the influence of an AC electric field. Next, various droplet unit operations such as transportation, extraction, and merging were demonstrated by the electrical tweezers. Finally, the electrical tweezer was used to demonstrate loop-mediated isothermal amplification (LAMP) of DNA.

### 4.1. Droplet Motion under Alternating Current (AC) Electric Field

**Figure 2:**
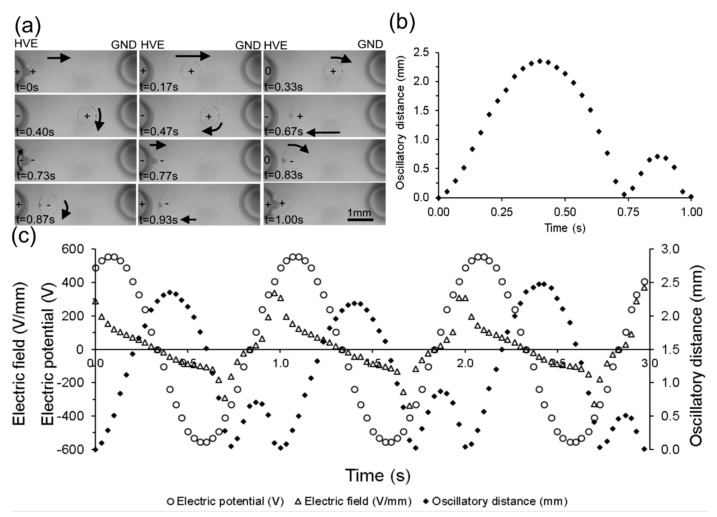
Droplet oscillatory motion with the high-voltage electrode under the influence of applied sinusoidal electric potential: (**a**) Sequential images of droplet motion with high-voltage electrode for one frequency cycle of applied potential of 560 V at 1 Hz; (**b**) oscillatory trip of droplet for the one frequency cycle of applied potential of 560 V at 1 Hz with the high-voltage electrode; (**c**) the droplet oscillatory motion synchronized with the applied sinusoidal voltage and electric field. Applied sinusoidal electric potential (○), sinusoidal electric field (Δ), Oscillatory distance (◊).

Experiments were conducted to characterize the effect of an applied alternating current (AC) electric field between the electrodes on the motion of the droplet. A droplet of deionized (DI) water (0.3 µL) was dispensed via a micropipette, at the interface of the dielectric medium of the immiscible bi-layer of oil, and positioned in between the fingers of the electrical tweezer (electrodes 4 mm apart). One electrode was connected to the high-voltage source terminal while the second was grounded. A sinusoidal potential with peak amplitude of 560 V and a frequency of 1 Hz was applied. The position of the droplet was recorded visually and is shown in Figure 2.

Under the influence of the electric field, the droplet was polarized and was attracted towards the high-voltage electrode. Upon contact (t = 0 s), the droplet acquired a charge (positive or negative depending on the polarity of the electrode at that instant in time). The charged droplet was then repelled from the electrode and moved away (t = 0.17 s) from it. The electric field imposed by the sinusoidal potential reversed (t = 0.33 s) before the droplet was able to reach the ground electrode, which changed the direction of the electrophoretic force, and attracted the droplet back (t = 0.47 s) to the high-voltage electrode, eventually contacting it at t = 0.73 s. Upon contact, the droplet once again acquired the charge of the electrode and was repelled from it, which resulted (t = 0.77 s) in a second, smaller oscillatory trip from the high-voltage electrode (at t = 1.0 s). During the larger oscillation trip, the droplet travelled the distance of 2.35 mm while, the maximum travelled distance was 0.71 mm during the shorter trip.

In order to understand the complex motion of the droplet in response to the applied voltage, the electric field was computed and plotted in relation with the position of the droplet. A two-dimensional numerical simulation (using COMSOL Multiphysics 5.1 software) was performed to calculate the electric field strength between the electrodes. The position of droplet during the oscillatory motion between electrodes, synchronized with the applied potential and the corresponding electric field strength between those electrodes is plotted with respect to time (Figure 2c).

Electric field strength between the electrodes for droplet position at any instance is plotted. The electric field strength at any particular location between the electrodes depends on the instantaneous applied voltage and its location between the electrodes. For instance, at t = 0 s droplet is in contact with the high-voltage electrode. The applied sinusoidal potential is ~490 V and electric field strength is ~288 V/mm at that instance. It can be seen that the motion of the droplet closely tracks the electric field intensity and its direction at that particular location where it is present. The droplet starts at the electrode surface where the electric field intensity is high and moves at high speed. As it moves away, the electric field intensity reduces both due to the change in the magnitude of the applied potential as well as due to the distribution of the field between the electrodes. Eventually, the electrical potential and the field change direction, which leads to a reversal in the direction of motion of the droplet. It arrives back at the electrode, makes contact, acquires charges once again and gets repelled from the electrode leading to the secondary motion. The secondary motion also follows the direction and the intensity of electric field. The synchronous plotting of the electric field and the position of the droplet demonstrates that the complex primary and a secondary oscillatory droplet motion is a result of temporal and spatial variation of the electric field encountered by the droplet.

### 4.2. Effect of Electric Field and Frequency

**Figure 3:**
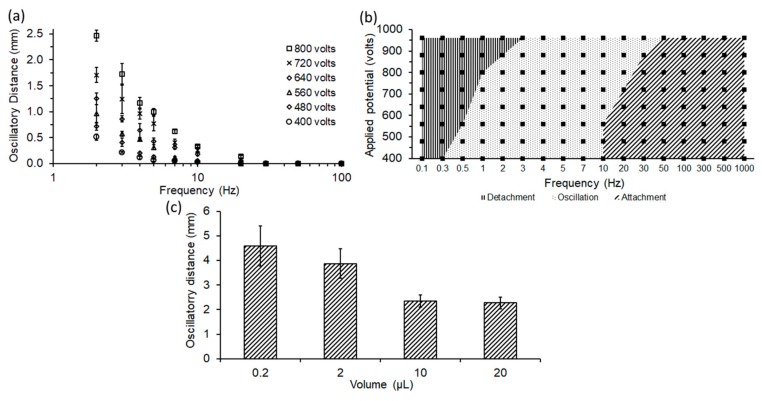
Characterization of water droplet (0.3 µL) motion with the high voltage electrode of electrical tweezer (electrodes 4 mm apart): (**a**) Maximum oscillatory distance of water droplet with the high-voltage electrode for tested range of frequencies (2–100 Hz) and for applied maximum sinusoidal electric potential (400–800 V); (**b**) electrical parameters to select the type of droplet motion with a high-voltage electrode under the tested range of frequencies (0.1–1000 Hz) and applied maximum sinusoidal electric potential (400–960 V); (**c**) dependence of oscillatory distance of water droplet with the high-voltage electrode for various droplet volumes (0.2, 2, 10, 20 µL), under the electric potential of 640 V (V/d = 160 V/mm) at 2 Hz.

The effect of sinusoidal applied potential and frequency on the maximum oscillatory distance travelled by the droplet was also characterized. For these experiments, a droplet (0.3 µL) was dispensed at the interface of bi-layer dielectric medium between the electrodes (4 mm apart). Various maximum sinusoidal electric potential of 400, 480, 560, 640, 720, 800 V (V/d = 100, 120, 140, 160, 180 and 200 V/mm) for the range of frequencies 2, 3, 4, 5, 7, 10 to 100 Hz were applied, and the movement of the droplet observed under those conditions. The resulting maximum distance of the droplet away from the high-voltage electrode is plotted against various field strengths, the frequency and is shown in Figure 3a.

It can be seen from these results that for any particular applied potential, the maximum oscillatory distance of droplet decreases with the increase in the frequency. For instance, at an applied potential of 800 V (V/d = 200 V/mm), the maximum oscillatory distance of droplet decreases from 2.47 to 0.33 mm due to an increase in frequency from 2 to 10 Hz. This reduction in distance can be largely attributed to the change in the duration that the charged droplet is exposed to the driving electric field within one cycle.

The maximum distance traveled also increases with the strength of applied potential. For instance, at 2 Hz, oscillatory distance increases from 0.51 to 2.47 mm with the increase in electric potential from 400 to 800 V (V/d = 100–200 V/mm). This increase can be attributed to not only the increase in the electric field strength, but also to the higher charges injected into the droplet as it contacts the high-voltage electrode. Since the electrophoretic force on the droplet is (F_e_ = Eq) a product of the electric field and the charge, the increase in electric field has a quadratic effect on the maximum distance traveled.

The behavior of the droplet under the influence of sinusoidal electric potential can be classified into three modes, namely: (a) Detachment, (b) Oscillation, and (c) Attachment (Figure 3b). Detachment of the droplet from the electrical tweezer occurs when the electric field is high, or the applied frequency is low such that the droplet is able to traverse the gap between the electrodes before the electric field is reversed. In this scenario, the droplet contacts the ground electrode, deposits the charge it acquired at the high-voltage electrode and becomes neutral. Subsequently, any motion of the electrode mechanically can detach the droplet due to inertia. For instance, in the case of a low electric potential of 400 V (V/d = 100 V/mm), the droplet was detached for the frequencies lower than 0.3 Hz. In contrast, for the higher electric potential of 960 V (V/d = 240 V/mm), the droplet remains attached until a frequency of 3 Hz is reached. Consequently, for the successful operation of electrical tweezers, detachment conditions need to be avoided. At reasonably high electric potential or frequencies, the droplet is in an oscillatory mode and bounces against the high-voltage electrode as the polarity of the electric field is reversed and before the droplet can reach the ground electrode within a cycle. Finally, at very high frequencies, the droplet cannot mechanically respond fast enough to the change in polarity of the applied potential and remains attached to the high-voltage electrode. The electric field then determines the strength of its attachment to the electrode and at high electric field, the droplet remains stuck even at a high speed of motion of the electrode. The oscillatory mode and the stationary modes are suitable for reliable transport of the droplet with the tweezer. Release of the droplet will be enabled in the detachment mode.

### 4.3. Effect of Volume

The volume of the droplet also has a significant role in determining the effective operating conditions for the tweezer. To determine this effect, droplets of different volumes (0.2, 2, 10 and 20 µL) were dispensed at the interface of the bi-layer dielectric medium between the electrodes (10 mm apart). The droplet behavior was tested for a frequency of 2 Hz, under the influence of electric potential of 1600 V (V/d = 160 V/mm). The oscillatory distance travelled by the droplet did change based on the size of the droplet, as shown in Figure 3c. The smaller droplets were found to travel over a larger distance, primarily due to their smaller inertial mass and faster response to the electrophoretic force. Therefore, the size of the droplet will have an effect that is similar to that of the electric field intensity in determining the various modes of operation of the tweezer.

## 5. Electrical Tweezers for Microfluidic Unit Operations

### 5.1. Droplet Transportation

**Figure 4:**
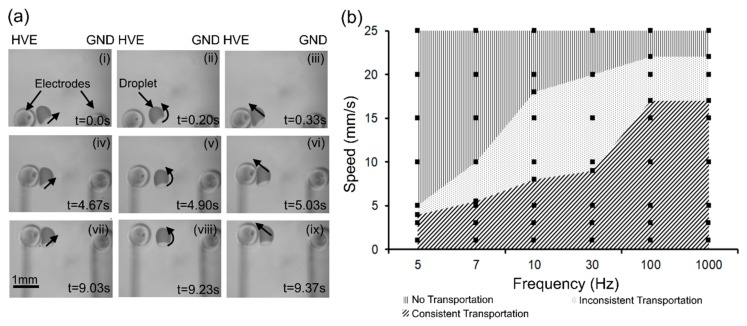
The utilization of electrical tweezer for the droplet transportation: (**a**) Sequential images (i–ix) illustrates the droplet transportation meanwhile the droplet is oscillating with the high-voltage electrode under the influence of electric potential of 480 V (V/d = 120 V/mm) at 3 Hz; (**b**) the characterization of droplet transportation with the electrical tweezer (electrodes 10 mm apart), under the influence of electric potential of 2000 V (V/d = 200 V/mm) at 5 Hz, is categorized into three regions; (i) no transportation, (ii) inconsistent transportation, and (iii) consistent transportation.

The electrical tweezer was used to transport a droplet in the bi-layer dielectric medium. In order to demonstrate this operation, an electric potential of 480 V (V/d = 120 V/mm) at 3 Hz was applied to an electrical tweezer (electrodes ~4 mm apart) with a water droplet (0.3 µL) in between it. The tweezer was moved at a speed of ~3 mm/s. The sequential images, shown in Figure 4a, demonstrate that the droplet oscillates with the high-voltage electrode (motion in the horizontal direction) while being simultaneously transported along with the electrode in the vertical direction.

In order to fully characterize the transport phenomena, the performance of the tweezer at various speeds of motion and frequencies of the electric field were analyzed. The electrical tweezer used electrodes 10 mm apart, was mounted on a DC powered motorized linear stage that was used to transport a droplet (5 µL) for fixed distance of ~20 cm at constant and defined velocities in the bi-layer dielectric medium. An electric potential of 2000 V (V/d = 200 V/mm) was applied and the frequency was varied from 5 to 1000 Hz. The results presented in Figure 4b show that both the speed and the frequency play a critical role and there are regions in the operating space that allow consistent transportation of droplet.

At low speeds (speed < 5 mm/s), the droplet can move along with the electrode consistently for all frequencies even in the oscillating regime. As the speed of the tweezer motion is increased, a higher frequency is required to ensure consistent transportation. For instance, at a speed of 15 mm/s, an applied frequency of 100 Hz is required to hold the droplet in the tweezer grip as it is being transported. A low frequency of 30 Hz results in inconsistent transportation where the droplet is sometimes attached to the tweezer and is transported, while in other instances, it is not. At low frequencies such as 7 Hz, the droplet is consistently left behind as the tweezer is moved.

The maximum speed with which the tweezers can be moved while still consistently transporting the droplet was found to be ~15 mm/s. Beyond 22 mm/s, the droplet is almost always left behind even at high frequencies beyond 1000 Hz. During transportation, the droplet experiences both attractive and repulsive forces. Electrophoretic forces of attraction serve to keep the droplet oscillating between the electrodes and transport it along with the tweezer, while inertia as well as drag forces on the droplet causes it to remain behind. When the electrophoretic force is significantly larger than the inertial and drag forces, the droplet tends to be consistently transported with the tweezer. When the frequency is low, the distance that the droplet travels away from the electrode (high field region) is higher and therefore the likelihood that it may be left behind by inertia is greater. On the other hand, at high frequencies, the distance traveled is smaller, the electrophoretic force encountered is higher, and therefore, the droplet is retained. At high speeds, the drag force becomes substantial and overcomes the electrophoretic force, dislodging the droplet from the high field region close to the electrode.

### 5.2. Droplet Extraction

The electrical tweezer was also used (electrodes 10 mm apart) to extract discrete water droplets from the outlet of the capillary tube positioned at the interface of the dielectric the bi-layer medium. In this experiment, a syringe pump was used to pump water (flow rate of 1 μL/min) to the tip of a capillary tube that was placed at the interface of the bi-layer oil medium. The tweezer was brought close to the emerging droplet at the capillary tip and the electrical potential from 0 to 6000 V (V/d = 0 to 600 V/mm) was applied. Next, the tweezer was moved forward (away from the capillary) to extract the droplet.

**Figure 5:**
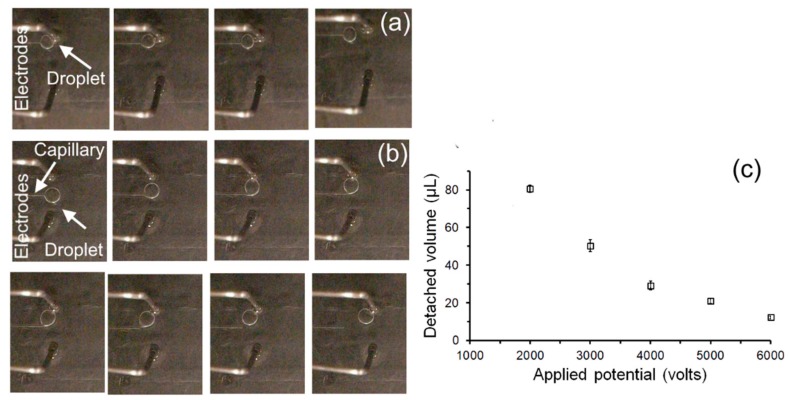
Extraction of a droplet from a capillary tip using electrical tweezer: (**a**) Sequential images of the movement of electrical tweezer close to the water droplet in the absence of an electric field; (**b**) sequential images of the extraction of droplet under the influence of electric potential of 2000 V (V/d = 200 V/mm) at 5 Hz; and (**c**) the dependence of detached droplet volume from the capillary on the applied sinusoidal electric field.

In the case when no electric field was applied (Figure 5a), the electrical tweezer was not able to dislodge the emerging droplet from the tip of the capillary even when physical contact between the electrode of the tweezer and the droplet was made. However, with an electric potential 2000 V of (V/d = 200 V/mm) at 5 Hz, the emerging droplet was first attracted to the high-voltage electrode and then moves with the electrode as it is moved away from the capillary (Figure 5b).

The volume of the extracted droplet was found to depend on the intensity of the applied electric potential. Surface tension forces between the droplet and the glass capillary should be overcome in order to detach the droplet. As the droplet grows in size beyond the size of the capillary, the droplet surface at the point of contact becomes tangential to the capillary tip. Therefore, the component of surface tension force at that interface along the axial direction of the capillary becomes smaller. This makes the force needed to dislodge the droplet becomes smaller and hence a lower field is required. (Figure 5c). Thus, a precise volume of liquid can be extracted by simply controlling the applied electric potential. This relationship may be related to the initial polarization of the droplet which is dependent on the electric field and the dielectrophoretic forces exerted on the droplet. The use of electric field to extract droplets provides an analog tunability to the volume that can be extracted. The capability to extract a defined volume can potentially be used to aliquot reagents and water to be combined in different ratios to produce varying concentrations.

### 5.3. Droplet Merging

**Figure 6:**
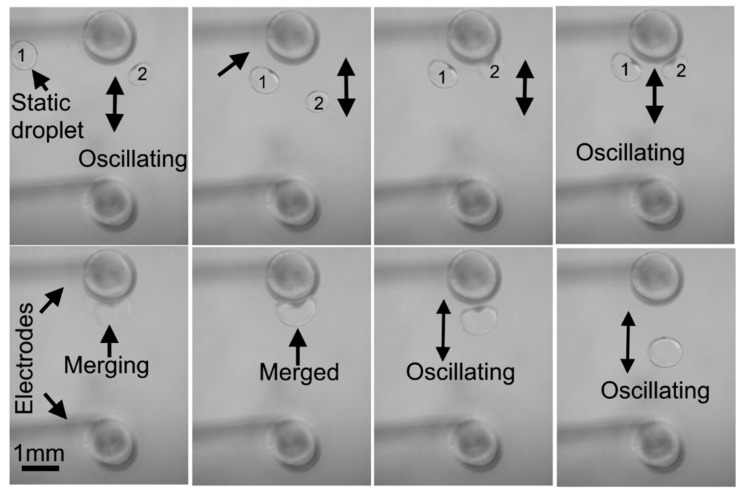
Sequential images of two droplets merging using electrical tweezers under the influence of applied potential of 560 volts (V/d = 140 V/mm) at 2 Hz.

Merging of droplets is crucial to mix reagents in microfluidics. To demonstrate droplets merging, an electric potential of 560 V (V/d = 140 V/mm) at 2 Hz was applied to the electrical tweezer (electrodes 4 mm apart). The first droplet (0.3 µL) was dispensed at the interface of the bi-layer dielectric medium between the electrodes, while the second droplet (0.3 µL) was positioned at the small distance (~5 cm) from the tweezers. The sequential images in Figure 6 demonstrate the merging of two droplets. It shows the tweezers transporting an oscillatory droplet to another static droplet and their eventual merging. The droplet (2) gets polarized under the influence of the electric potential and acquires charge by contact with the high-voltage electrode.

The charged droplet starts oscillatory motion with the high-voltage electrode under the influence of electric field. Next, the oscillatory charged droplet was transported by the electrical tweezers to the vicinity of the stationary droplet (1).

As the tweezer approached Droplet (1), it also becomes polarized and was attracted towards the high-voltage electrode. Upon making a direct contact, it attains a charge from the high-voltage electrode and begins an oscillatory motion with the same electrode. Both droplets immediately fuse together while performing the oscillatory motion and the merged droplet continues its oscillatory motion with the high-voltage electrode. The time required to merge depends on the initial positions of stationary and approaching droplets. In all cases the droplets merge within 2–3 oscillations with the electrodes when they approach the electrode and are in the vicinity of each other. These experiments demonstrate that not only can the individual droplets be transported, but they can also be merged into a single droplet. The oscillatory motion of the droplet, subsequent to merging, causes complete mixing.

## 6. DNA Analysis through Amplification

In order to demonstrate the utility of the electrical tweezer in performing unit operations for a biochemical assay, DNA amplification using the loop-mediated isothermal amplification (LAMP) method [29] was performed. The electric potential of 1200 V (V/d = 120 V/mm) at 10 Hz was applied to the electrical tweezer (electrodes 10 mm apart) to hold, transport and merge the droplets containing the LAMP reagents and the DNA sample.

**Figure 7:**
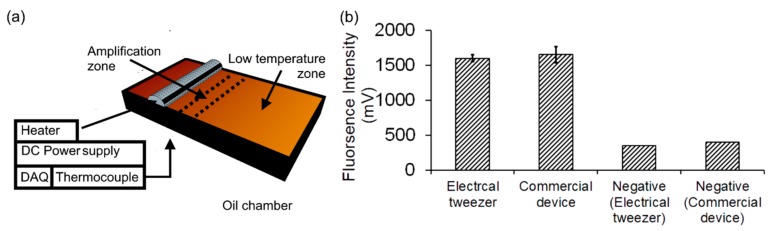
Loop-mediated isothermal amplification of DNA by electrical tweezers: (**a**) Schematics of bi-layer oil chamber for DNA amplification; (**b**) comparison of end- point fluorescence intensity of positive samples by electrical tweezers and fluorescence reader (ESE-Quant Tube scanner) with the negative control at ~65 °C.

First, the droplet of the reaction mixture (20 µL); Optigene isothermal master mix (15 µL) and primer mix (5 µL), was dispensed at the interface of bi-layer dielectric medium in the low-temperature zone of the oil chamber (Figure 7a). First, electrical tweezer was used to manually transport the droplet (20 µL) to the second droplet of DI water (5 µL) that was positioned in the low temperature zone, to perform negative reaction. Upon approach, the droplet with reaction mixture and the droplet with DI water merged with each other. Then, the merged droplet (25 µL) was transported and positioned in the amplification zone (~65 °C) for 30 min. Amplification using negative samples were performed at first so as to avoid any potential contamination effects. Then, for the positive reactions, water droplet was replaced by the droplet (5 µL) containing the DNA template (10^8^ DNA copies/droplet). Finally, the droplets with the amplified sample were extracted using a pipette and loaded in the commercial fluorescence reader (ESE-Quant Tube scanner, Qiagen, Hilden, Germany) to detect amplification. Two control amplification reactions of same volume of reagents, one with DNA template and second with DI water, were performed in the commercial fluorescence reader (ESE-Quant Tube scanner, Qiagen, Hilden, Germany). The results for three replicates of the positive sample in each case are demonstrated and compared with negative samples, as shown in Figure 7b. It can be seen that the amplification in the droplet format using the electrical tweezers had a similar performance as the conventional device. The positive samples were also significantly (~5 times) different from the negative control, both in the commercial device as well as when using the electrical tweezer.

## 7. Conclusions

In conclusion, we have successfully demonstrated a simple approach to handle single or multiple droplets to perform biochemical assays at low throughput. This approach uses an electrical tweezer to handle and position the droplets and a bi-layer dielectric medium to confine the droplets in a single plane for easy handling. Although the characterization of the tweezers was performed using a mechanized stage, it is designed to be manually control by the user making is easy and intuitive to use. The motion of a single droplet under the influence of a sinusoidal electric field was studied. Based on this knowledge, various unit operations required such as droplet transportation, extraction, aliquoting and merging were demonstrated and the optimal operating conditions for them, determined. Finally, these operations were utilized to successfully perform isothermal amplification of DNA. Current format supports cleaning or replacing the low-cost needles that are used as a tweezer. Electrical conditions that will promote self-cleaning or prevent cross-contamination can be explored in the future to reuse them over longer durations of time. This approach is expected to be of broad applicability in other biochemical assays such as immunoassays, and cell-based assays as well as in small-organism-based assays.
